# Postural Balance in Italian Air Force Pilots: Development of Specific Normative Values

**DOI:** 10.3390/audiolres15030070

**Published:** 2025-06-12

**Authors:** Vincenzo Fiorillo, Barbara Martino, Valeria Castelli, Eliana Filipponi, Leonardo Braga, Alessandro Randolfi, Emanuele Garzia, Federica Di Berardino

**Affiliations:** 1 Otolaryngology Unit, Aerospace Medicine Institute “A. Mosso”, Italian Air Force, 20138 Milan, Italy; 2 Department of Clinical Sciences and Community Health, University of Milan, 20122 Milan, Italy; 3 Direzione Aziendale Professioni Sanitarie, Fondazione IRCCS Ca’ Granda Ospedale Maggiore Policlinico, 20122 Milan, Italyeliana.filipponi@unimi.it (E.F.); 4 Laboratory of Healthcare Research & Pharmacoepidemiology, Department of Statistics and Quantitative Methods, University of Milano-Bicocca, 20126 Milan, Italy; l.braga1@campus.unimib.it; 5 Aerospace Medicine Institute “A. Mosso”, Italian Air Force, 20138 Milan, Italy; 6 Audiology Unit, Department of Surgical Sciences, Fondazione IRCCS Ca’ Granda Ospedale Maggiore Policlinico, 20122 Milan, Italy

**Keywords:** sensory organization test, normal value in army aviators, equilibrium evaluation, return to duty after balance injury

## Abstract

**Objectives**: Assessing balance in highly trained individuals, such as military pilots, poses challenges, as deficits may be underestimated when compared to general population norms. To address this, several studies have proposed tailored databases providing reference values for specific populations. This study retrospectively analyzed balance characteristics in active-duty military pilots of the Italian Air Force. **Methods**: We enrolled 106 subjects split into two groups: 53 military pilots from the Italian Air Force and 53 civilians without flight experience or exposure to specific vestibular stimuli. All participants underwent ENT examinations with audiometric testing to exclude related pathologies, followed by a personal history collection. Subsequently, they completed the EquiTest protocol across six standard conditions. **Results**: Significant differences were observed between Army Aviators and Non-Aviators. The PREF variable showed the most consistent distinction, with military pilots demonstrating a superior performance (*p* < 0.01). Additionally, borderline differences were noted in Condition 6 of the equilibrium scores (*p* = 0.056), and in the Centre of Gravity (COG) analysis along the X-axis for Conditions 1 and 5 (*p* = 0.090), and for Condition 2 (*p* = 0.050). These findings suggest enhanced postural control strategies among Army Aviators under conditions of sensory conflict. **Conclusions**: These findings suggest that normative balance values specific to military pilots should be used when evaluating aviators recovering from balance deficits. Such tailored benchmarks can help determine the need for rehabilitation before returning to duty, ensuring optimal performance under demanding conditions. Further research is necessary to explore the underlying mechanisms responsible for these adaptations and to identify the specific stimuli that contribute to the enhanced balance capabilities observed in this highly trained population.

## 1. Introduction

The evolution of humans from quadrupedalism to bipedalism has led to the development of a highly sophisticated system responsible for maintaining an upright posture, involving the interaction of multiple, closely interconnected systems. Postural stability, a crucial aspect of human motor control, is maintained by three primary systems: the visual, vestibular, and proprioceptive systems. These systems work synergistically, transmitting continuous information to the central nervous system (CNS), which integrates the input to generate both reflexive and cortically mediated responses. The mechanisms governing postural stability and control include supraspinal processes for anticipatory postural adjustments, cortical active control, and reflex pathways, all based on input from peripheral receptors [1].

Individuals performing at high levels, such as military personnel and athletes, demonstrate an enhanced ability to adapt their postural control system, improving stability even in challenging and non-standard conditions [2,3,4,5]. In the specific context of pilots, it becomes imperative to employ instrumental methods capable of separately evaluating the different sensory systems contributing to balance, always considering the multifactorial nature of overall homeostasis.

Currently, the Sensory Organization Test (SOT) is considered the gold standard for assessing postural stability [6]. This test evaluates how the CNS integrates and prioritizes sensory input from the visual, vestibular, and somatosensory systems. While widely used in clinical settings for assessing patients recovering from neurosensory injuries—particularly concussion and traumatic brain injury—recent research has increasingly focused on the SOT’s application in military contexts, particularly in developing normative databases tailored to specific occupational groups [2,5,7].

Dynamic posturography, exemplified by the SOT, offers an objective evaluation by systematically distorting sensory inputs, thereby assessing the individual’s capacity to compensate. The SOT allows for the calculation of the Center of Gravity (COG) and its stability limits, while quantitatively and qualitatively analyzing different balance strategies, such as ankle or hip dominance. Importantly, the SOT should not be viewed as a diagnostic tool for deficits, but rather as an assessment of functional abilities. It also identifies the patient’s preferred sensory system for maintaining balance and evaluates the efficiency of alternative systems [6,8].

In recent years, several normative databases have been developed, identifying threshold values specific to various populations, including children, elderly individuals, patients with vestibular disorders, and military personnel [2,3,4,5,9,10,11,12]. It is crucial to avoid using generic normative values for specialized groups, as their postural control capabilities may differ significantly. Indeed, even when scores fall within standard ranges, specific rehabilitation programs may be necessary before a patient—or in this case, a pilot—can safely return to duty. Recently, a study focusing on military rotary-wing pilots using the SOT aimed to define actual normative values specific to this population, which is continually trained to manage balance in scenarios not typical of everyday life [5]. Inspired by this valuable work, we decided to expand it by increasing the sample size and including additional subcategories.

The primary objective of our study is to define precise normative values for highly trained military personnel, categorized by the type of aircraft piloted, and compare these values to those of a civilian control group, following the approach suggested by Baylor et al. in 1992 [3]. Moreover, we aim to explore how training on specific aircraft influences balance performance. However, for military personnel not subjected to such high-level training, the manufacturer’s normative values remain appropriate for postural assessment [7]. Therefore, it becomes essential to establish new normative thresholds tailored to each cohort, ensuring an accurate evaluation of postural deficits and determining when pilots can safely resume flying, or if additional balance rehabilitation is warranted [2,3,5].

## 2. Materials and Methods

### 2.1. Participants

A total of 106 individuals participated in our study, divided into two groups: Army-trained aviators and non-aviators. The non-aviator group consisted of flight applicants who had not yet flown and had no exposure to specific vestibular stimuli. Each group included 53 participants.

The age range in the Army aviators’ group was from 22 to 55 years (mean 40 ± 9 years), while the civilian group ranged from 18 to 66 years (mean 30 ± 12 years).

Only male participants were included in this study, as the current number of female pilots in the Air Force remains insufficient to allow for reliable statistical comparisons. This decision was made to minimize potential biases and ensure the accuracy of the statistical analysis.

A comprehensive list of the inclusion and exclusion criteria applied in the study is provided in Table 1.

### 2.2. Instrumentation

The SOT was delivered using NeuroCom SMART EquiTest Clinical Research System (Natus Medical international, Clackamas, OR, USA) with the Data Acquisition Toolkit (version 9.2). The SMART EquiTest includes an 18″ × 18″ dual force plate and a visual surround, which withers are fixed or moveable (rotating up/down or forward) in reference to the participant’s sway.

### 2.3. Procedures

At the beginning of the evaluation, each participant underwent a comprehensive Ear, Nose, and Throat (ENT) examination, which included otoscopy, rhinoscopy, and oroscopy, to rule out any ENT-related pathologies. In addition, pure-tone audiometry (Clinical Audiometer Piano, Inventis S.r.l., Padova, Italy) was performed at frequencies ranging from 250 Hz to 8000 Hz (250-500-1000-2000-3000-4000-6000-8000 Hz). In addition, a bedside examination was conducted to evaluate vestibular function, and no indications were found to warrant further second-level diagnostic investigations.

Prior to the start of the test, our team provided each participant with a detailed explanation of the procedure. Participants were required to remove their shoes, although they were given the option to retain their socks if preferred. Once positioned on the platform, each participant was secured with a safety harness to minimize the risk of falls during the test.

Within the EquiTest system, the patient undergoes six different conditions: conditions 1, 2, and 3 with a fixed platform, and conditions 4, 5, and 6 with a sway-referenced platform. In the sway-referenced conditions, the platform moves in relation to the patient’s sway to minimize proprioceptive feedback alterations. This platform adjustment is called “sway-referenced motion”. The SOT aims to isolate the contribution of each sensory system. In Conditions 1 and 2, the platform and visual surround are fixed, with the participant standing first with their eyes open (Condition 1) and then with their eyes closed (Condition 2). In Condition 3, the support surface remains fixed, but a sway-referenced visual surround is introduced, highlighting the role of visual input. Condition 4 evaluates the combination of visual and somatosensory information, while conditions 5 and 6 isolate the vestibular system’s contribution [6,9]. Throughout the test, participants were instructed to look straight ahead, remain calm, keep their arms relaxed at their sides, and maintain their feet firmly on the platform. In accordance with the protocol, certain conditions were performed with eyes closed.

Each condition was repeated twice, with approximately one minute of rest between trials. The rest period between different conditions was about 20 s. Any loss of balance during a trial, including contact with the surrounding structure (e.g., walls), was recorded as a fall. The six conditions of the Sensory Organization Test (SOT) protocol are summarized in Table 2.

Thanks to the data obtained from the different conditions, it was possible to perform the Sensory Analysis Ratio using Cevette’s formula, as presented in Table 3. Cevette’s formula provides a method for quantifying the contribution of each system to postural control. The SOM Ratio assesses the subject’s ability to rely on somatosensory input to maintain balance when visual input is absent (eyes closed on a stable surface). Condition 1 serves as the baseline with all sensory inputs available. The VIS Ratio evaluates the effectiveness of the visual system in maintaining balance when somatosensory input is altered (eyes open on a sway-referenced surface). The VEST Ratio measures the reliance on vestibular input when both visual and somatosensory inputs are removed or altered (eyes closed on a sway-referenced surface). Finally, the PREF Ratio reflects the subject’s tendency to depend on visual information, even when it is inaccurate or conflicting. Conditions 3 and 6 introduce visual conflict (sway-referenced vision), while Conditions 2 and 5 provide accurate non-visual sensory information. Ratios close to 1 indicate an effective use of the corresponding sensory system, while lower ratios suggest a dysfunction or over-reliance on alternative sensory pathways.

The study was conducted in accordance with the Strengthening the Reporting of Observational Studies in Epidemiology (STROBE) Statement: guidelines for reporting observational studies and with the Helsinki Declaration. The study was performed under the Milan University Research Ethics Board guidelines for retrospective observational study on healthy subjects. No formal research ethics board approval was necessary and therefore no reference number was generated. The participation in the study was voluntary and the patients were not paid for it. Informed consent was obtained from all subjects involved in the study. The patients’ anonymity has been guaranteed. 

### 2.4. Statistical Analysis

We assigned an identification number to each subject enrolled in the study sample in accordance with current regulation for handling personal data. We analyzed the collected data using, firstly, simple descriptive statics. Due to the small sample size, we employed a non-parametric test.

At the beginning, we verified the assumption of normality for the groups involved in different comparisons. Once confirmed, we applied the *t*-test to compare two groups. Conversely, in case this assumption was not met, we applied the Wilcoxon Test. Finally, for comparisons between values in Trial 1 and Trail 2, we employed a *t*-test for paired groups.

We considered a *p*-value < 0.05 as statistically significant. The statistical analyzes were conducted using the R Studio statistical software (version 4.3.1).

## 3. Results

The results of the study are reported in the following tables.

In Table 4, we summarized the aviator group (group 1 = 53 patients), dividing participants based on the type of aircraft piloted—either rotary-wing or fixed-wing aircraft. For fixed-wing aircraft, we further specified whether participants operated jet or transport planes (applicable only to group 1, i.e., the aviators’ group).

**Figure 1:**
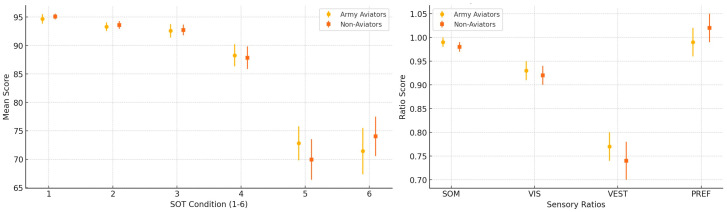
The equilibrium score (ES) with medium values for each condition and Sensory Analysis Ratio and DS with a 95% confidence interval.

**Figure 2:**
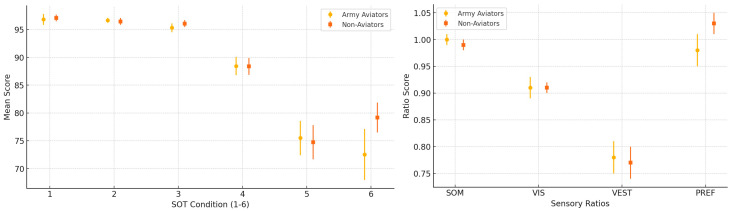
The strategy with medium values for each condition and Sensory Analysis Ratio and DS with a 95% confidence interval.

**Figure 3:**
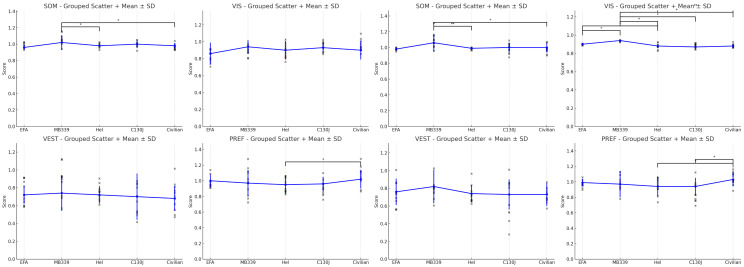
The equilibrium score and strategy with medium values for Sensory Analysis Ratio in Trial 1. The asterisk symbol is located next to statistically significant values in the figure. In the figure, a double asterisk symbol indicates values approaching the threshold of statistical significance.

**Figure 4:**
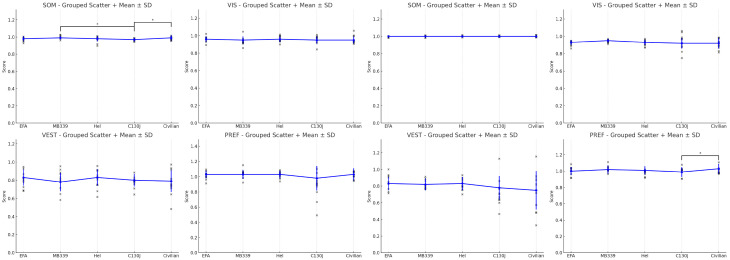
The equilibrium score and strategy with medium values for Sensory Analysis Ratio in Trial 2. The asterisk symbol is located next to statistically significant values in the figure.

In the following figures, from 1 to 4 [Figure 1, Figure 2, Figure 3 and Figure 4], additional results are summarized. We used graphs as they are more accessible than Table 5, Table 6, Table 7, Table 8 and Table 9, which could have been confusing due to the volume of data they contain.

## 4. Discussion

The SOT is widely used in many otoneurologic centers for both the diagnosis and rehabilitation of individuals with balance disorders [8,12,13,14]. It allows for the quantification of the influence of sensory afferents and, consequently, facilitates the planning of rehabilitation strategies or the evaluation of improvements over time [14]. More recently, increasing attention has been placed on defining specific reference values for particular population cohorts that differ from the general population due to factors such as pathology, specialized training, or aging [13,15]. Several studies have focused on evaluating distinct populations, including pediatric patients, migraine sufferers, individuals with mild traumatic brain injury, military personnel, gymnasts, and others. As a result, normative values tailored to these unique populations have begun to emerge [2,3,4,5,7,8,11].

As early as 1992, Baylor KA et al. hypothesized that military pilots, due to the intrinsic characteristics of their daily activities, might exhibit variations in postural control features compared to the general population [3]. Despite this, the literature still contains relatively few studies addressing the specific postural capabilities of pilots, who consistently demonstrate balance characteristics significantly above average. More recently, a study focusing on military rotary-wing pilots using the SOT aimed to define actual normative values specific to this population, which is continually trained to manage balance in scenarios not typical of everyday life [5].

In our study, enrolled pilots included not only rotary-wing pilots but also fixed-wing pilots operating transport and jet aircraft. Simultaneously, we recruited a group of Non-Aviators as a control group, allowing us to avoid relying solely on normative data provided by the manufacturer. To ensure a more comprehensive evaluation, we also included audiometric testing alongside the SOT to rule out any differences attributable solely to cochlear issues.

At the conclusion of the study, as detailed in the ‘Results’ section, we observed statistically significant differences in military pilots’ performance compared to the general population. Notably, in Strategy Condition 6 of the Sensory Organization Test, Army Aviators exhibited a lower mean equilibrium score (72.53 ± 17.02) compared to Non-Aviators (79.16 ± 9.94), with the difference approaching statistical significance (*p* = 0.056). Condition 6 represents the most challenging scenario, in which both visual and proprioceptive inputs are unreliable, and vestibular function becomes the primary contributor to balance. The trend observed suggests that, under extreme sensory conflict, Non-Aviators may have performed slightly better than Army Aviators, although the difference did not reach the conventional threshold for statistical significance. This borderline result may warrant further investigation in larger cohorts to elucidate whether it represents a consistent pattern or sample variability. In a similar manner, Figure 3 illustrates a further accentuation of this difference, mirroring the trend previously observed in Condition 6 and is particularly evident in the PREF variable in strategy (*p* < 0.01). This score reflects the ability to maintain balance when visual inputs become unreliable—specifically when visual cues are distorted, and the visual environment conflicts with information from other sensory systems [6,9]. These findings highlight the greater adaptability and resources available to military pilots, which can be attributed to their specialized training. Although no significant differences were found in the individual conditions or scores, the PREF variable was consistently distinct in both equilibrium and strategy scores, indicating that military pilots experience fewer oscillations and manage postural control more effectively when visual input is compromised.

As a secondary outcome, we examined differences between the first and second trials to evaluate rapid learning and adaptation. Within the Army group, pilots were categorized based on the type of aircraft operated. Notably, significant improvement was observed across all subgroups between trials, indicating rapid adaptation, except among jet pilots. However, it is important to note that the jet pilots had already demonstrated a very high baseline performance, leaving limited room for significant improvement. This learning effect was also evident among civilians, even in simple conditions such as Condition 1, suggesting that emotional factors (e.g., anxiety during the first trial) may influence performance [16,17].

The third outcome is the analysis of the Centre of Gravity (COG) median values along the X (lateral) and Y (anterior–posterior) axes and it revealed limited but noteworthy trends between Army Aviators and Non-Aviators. Although no strongly significant differences were detected, borderline values were observed for Xmed in Conditions 1 and 5 (*p* = 0.090) and reached near statistical significance in Condition 2 (*p* = 0.050). For Ymed, a similar borderline difference was noted in Condition 1 (*p* = 0.058). These findings suggest that Army Aviators may adopt slightly different postural control strategies, potentially involving an increased lateral displacement or forward positioning of the COG under certain testing conditions. This may reflect profession-specific adaptations related to the demands of flight, where the anticipatory activation of stabilizing muscle groups could contribute to subtle shifts in balance control.

### Subgroup Performance

Finally, we compared the performances of the various subgroups, both among themselves and against the civilian group. Interestingly, it was not the equilibrium scores but rather the strategy scores that revealed the most marked differences. C130J pilots demonstrated characteristics closely resembling those of civilians and, in some cases, even slightly lower values. Conversely, jet and MB339 pilots stood out with a superior performance, as expected, while rotary-wing pilots exhibited unique characteristics. Specifically, for rotary-wing pilots, the PREF variable in the strategy scores showed a reversal: civilians had higher values, with a statistically significant difference (*p* < 0.01). This suggests that the observed differences are likely related to the specific type of aircraft flown.

Given these findings, we hypothesize that jet pilots possess an exceptionally high balance performance because their systems are continuously trained to handle sensory mismatches between visual, vestibular, and proprioceptive inputs [18,19,20]. MB339 pilots, though primarily visual flight operators, share a background in jet piloting, which may explain their ability to reactivate previously developed strategies and improve significantly in the second trial [18]. Moreover, military personnel are systematically trained to perform under physical and emotional stressors, including sleep deprivation, situational awareness, and dehydration. As previous studies on PPPD have demonstrated the influence of emotional factors on postural control, it is plausible that pilots develop strategies to minimize emotional interference and rapidly adjust to unexpected conditions [21]. These results are summarized in Figure 1 and Figure 2, which display the inter-group comparisons of equilibrium scores and Sensory Analysis Ratios for both trials.

Helicopter pilots, meanwhile, face different stressors but are highly reliant on visual cues. Their performance indicates a strong ability to selectively utilize different sensory systems, although their visual dependence may be even greater than that of civilians—possibly due to an increased visual engagement during flight. It is plausible that helicopter pilots, who are frequently exposed to complex visual stimuli and are trained predominantly for visual flight rather than instrument flight, have developed specialized integrative systems allowing rapid postural adjustments. Further studies examining their gaze behavior would be valuable to confirm this hypothesis [22]. Additionally, it is worth considering the link between SOT performance and utricular function, as rotary-wing pilots are primarily exposed to rotational rather than vertical accelerations, unlike jet pilots [23].

Finally, regarding C130J pilots, our data suggest that, while they demonstrate better balance capabilities than the general population (as indicated by standard normative values), their performance is not as exceptional as their colleagues operating other aircraft. This is likely due to the absence of specific stimuli such as angular or linear accelerations during flight.

## 5. Limits

The main limitations of this study are primarily related to the sample size of the subgroups. In particular, it would be advisable to increase the number of fighter jet pilots to establish more precise and reliable reference values specific to this category. Another limitation concerns the vestibular assessment, which was based exclusively on posturography. A more comprehensive evaluation should include additional tests, such as the assessment of the Vestibulo–Ocular Reflex (VOR) using the Video Head Impulse Test (vHIT), ocular movement analysis, and Visual Vertical Subjective (SVV). These additional evaluations would help to better understand the potential factors contributing to the superior balance performance observed in army-trained pilots compared to the civilian population. Moreover, the SOT represents an artificial assessment that differs from real-world flight conditions. In the future, newer force platforms, potentially combined with virtual reality technologies, may provide a more accurate simulation of real-life scenarios and enhance ecological validity.

## 6. Conclusions

The primary aim of this study was to define tailored normative values for trained military pilots and their respective subgroups, avoiding exclusive reliance on manufacturer-provided reference data. These reference values may also serve as a useful tool for rehabilitation or retraining programs for pilots recovering from vestibular or neurological impairments, particularly considering that the EquiTest system can be deployed in combat environments as well [24,25,26,27].

The objective was to define appropriate normative values specific to military personnel, particularly pilots, to guide decision-making before returning them to duty following balance-compromising events, as is commonly done for other specialized populations [24,28,29,30,31]. Our findings suggest the necessity of implementing normative values tailored to each type of aircraft. Furthermore, it would be beneficial to employ these reference values in conjunction with additional functional tests to assess pilots’ postural performance comprehensively. This approach would also facilitate the development of individualized rehabilitation protocols aimed at optimizing recovery and maintaining operational readiness in these hyper-trained populations.

## Figures and Tables

**Table 1:** Inclusion and exclusion criteria.

Exclusion Criteria	Inclusion Criteria
Dizziness or motion sickness	Male
Unable to move head right or left	Age < 18 years
Head injury	Italian air force pilot in service (group 1)
Neurological injury (MS, VS)	No-flight people (group 2)
Migraine	
Diabetes	
Any otological pathology or surgery (MeD, Col, etc…)	
Any ear surgery	
Any vestibular deficit (included otolithic vertigo)	
Major cerebrovascular disorder	
Unsolved neck injury or whiplash	
Systemic disorder (CRF, cirrhosis, MD, …)	
Substance use (cannabis, cocaine, etc.)	
SSNL	

MS: multiple sclerosis, VS: vestibular schwannoma, SSNL: sudden sensorineural hearing loss, MeD, Meniérè’s disease, CRF: chronic renal failure, Col: colestheatoma, MD, mellitus diabetes.

**Table 2:** Characteristics of SOT conditions [6,9].

Condition	Vision	Surface (Platform)	Visual Surround	Sensory System Used
1	Eyes open	Stable	Stable	Somatosensory
2	Eyes closed	Stable	NA	Somatosensory
3	Eyes open	Stable	Sway-referenced *	Somatosensory
4	Eyes open	Sway-referenced *	Stable	Vision
5	Eyes closed	Sway-referenced *	NA	Vestibular
6	Eyes open	Sway-referenced *	Sway-referenced *	Vestibular

NA: Not applicable. * The surface or visual surround moves in accordance with each individual’s anterior–posterior center of pressure sway.

**Table 3:** Sensory Analysis Ratio [6,9].

Somatosensory [SOM]	$\frac{Condition\;2}{Condition\;1}$
Visual [VIS]	$\frac{Condition\;4}{Condition\;1}$
Vestibular [VIS]	$\frac{Condition\;5}{Condition\;1}$
Preference for visual information [PREF]	$\frac{Condition\;3+Condition\;6\;}{Condition\;2+Condition\;5}$

**Table 4:** Participants of group 1 divided by type of aircraft.

Aircraft	Number of Patients
C130J	9
MB339	11
Hel	28
Eurofighter Typhoon	5

**Table 5:** Audiological results (hearing loss as defined by Goodman, modified by Clark) [10].

	Normoacusis	Hearing Loss			
		Mild	Medium	Severe	Profound
MB339	9	2	0	0	0
Jet	5	0	0	0	0
Helicopter	15	10	3	0	0
C130J	7	2	0	0	0
Civilian	52	1	0	0	0

**Table 6:** The equilibrium score (ES) with medium values for each condition and Sensory Analysis Ratio.

Medium	Army Aviator		Non-Aviator		*p*-Value
	M	SD	M	SD	
1	94.68	3.14	95.09	1.86	0.540
2	93.29	2.78	93.61	2.56	0.450
3	92.59	4.42	92.73	3.45	0.710
4	88.26	7.22	87.86	7.31	0.640
5	72.81	11.02	69.96	13.21	0.340
6	71.44	15.12	74.03	12.80	0.200
SOM	0.99	0.04	0.98	0.03	0.680
VIS	0.93	0.08	0.92	0.08	0.450
VEST	0.77	0.12	0.74	0.14	0.230
PREF	0.99	0.10	1.02	0.10	0.020 *

The asterisk symbol is located next to statistically significant values in the table.

**Table 7:** The strategy with medium values for each condition and Sensory Analysis Ratio.

Medium	Army Aviator		Non-Aviator		*p*-Value
	M	SD	M	SD	
1	96.83	3.59	97.08	2.18	0.620
2	96.65	1.61	96.45	2.32	0.760
3	95.32	2.95	96.08	2.23	0.310
4	88.44	6.08	88.37	5.62	0.590
5	75.50	11.46	74.74	11.37	0.670
6	72.53	17.02	79.16	9.94	0.056 **
SOM	1.00	0.05	0.99	0.03	0.970
VIS	0.91	0.06	0.91	0.05	0.310
VEST	0.78	0.12	0.77	0.11	0.580
PREF	0.98	0.10	1.03	0.08	<0.010 *

The asterisk symbol is located next to statistically significant values in the table. In the table, a double asterisk symbol indicates values approaching the threshold of statistical significance.

**Table 8:** The Centre of Gravity (COG) with medium values.

Xmed	Army Aviator		Non-Aviator		*p*-Value	Ymed	Army Aviator		Non-Aviator		*p*-Value
	M	SD	M	SD			M	SD	M	SD	
1	0.30	0.43	0.11	0.43	0.090	1	0.15	0.01	−0.10	0.01	0.058 *
2	0.29	0.43	0.11	0.49	0.050 *	2	0.12	0.81	0.08	0.71	0.690
3	0.25	0.43	0.15	0.43	0.400	3	0.10	0.81	−0.02	0.69	0.360
4	0.27	0.50	0.17	0.38	0.390	4	−0.03	0.78	−0.16	0.78	0.250
5	0.31	0.53	0.13	0.49	0.090	5	0.10	1.03	0.01	0.84	0.970
6	0.21	0.48	0.11	0.43	0.290	6	0.06	1.14	−0.08	0.89	0.720

The asterisk symbol is located next to statistically significant values in the table.

**Table 9:** Normative values. NV: Natus’ values. DNV: Developed normative values.

	NV	DNV
Condition		
1	90	89.5
2	85	89.6
3	86	85.4
4	70	80.6
5	52	55.6
6	48	54.5
SOM	94	95
VIS	77,7	85
VEST	57,7	60
PREF	97,8	88

## Data Availability

The original contributions presented in this study are included in the article.
